# Demonstration of a Flexible Graphene-Based Biosensor for Sensitive and Rapid Detection of Ovarian Cancer Cells

**DOI:** 10.1186/s11671-021-03633-9

**Published:** 2021-12-23

**Authors:** Ling Han, Qi Wan, Ai Zheng, Yunchuan Guo, Yali Chen

**Affiliations:** 1 Department of Gynecology and Obstetrics, West China Second Hospital, Sichuan University, Chengdu, 610041 People’s Republic of China; 2 Key Laboratory of Birth Defects and Related Diseases of Women and Children (Sichuan University), Ministry of Education, Chengdu, 610041 People’s Republic of China; 3 Chengdu Ginkgo Electronics Technology Co., Ltd., Chengdu, 610213 People’s Republic of China

**Keywords:** Flexible graphene-based biosensor, Circulating tumor cell (CTC), Ovarian cancer

## Abstract

It is significant to develop an efficient early detection and prediction method for ovarian cancer via a facile and low-cost approach. To address such issues, herein, we develop a novel circulating tumor cell (CTC) detection method to sensitively detect ovarian cancer by using a flexible graphene-based biosensor on polyethylene terephthalate (PET) substrate. The results show that the graphene-based flexible biosensor demonstrates sensitive and rapid detection for ovarian cancer cells: it delivers obvious different responses for cell culture medium and cancer solution, different cancer cells and cancer cell solution with different concentrations; it demonstrates high sensitivity for detecting several tens of ovarian cancer cells per ml; moreover, the flexible graphene biosensor is very suitable for rapid and sensitive detection of ovarian cancer cells within 5 s. This work provides a low-cost and facile graphene biosensor fabrication strategy to sensitively and rapidly detect / identify CTC ovarian cancer cells.

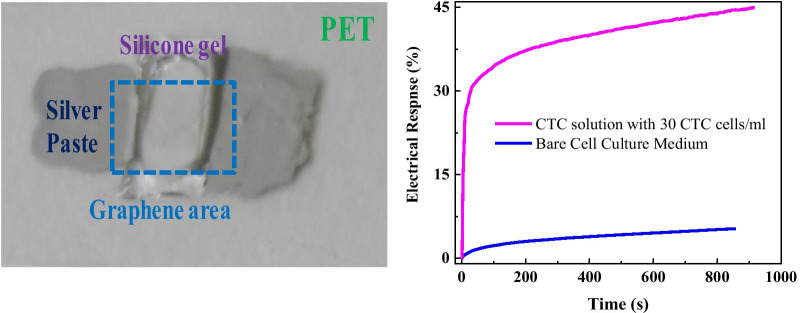

## Introduction

Ovarian cancer is the second most common gynecological cancer and has the highest mortality among the gynecological cancers [1, 2]. So far, the ovarian cancer patients are generally diagnosed very late due to the nonspecific symptoms of ovarian cancer and the lack of effective early screening methods. Imaging combined with carbohydrate antigen CA125 can be used in detection, diagnosing recurrences after surgery or chemotherapy. CA125 is not a single accurate marker for ovarian cancer because it is affected by numerous factors and has high false positive predictive value. The sensitivity of elevated CA125 (> 35 U/mL) which we used in the diagnosis of the recurrence of ovarian cancer is less than 70% [3]. Ultrasound examination and radiological examination also have no adequate sensitivity nor specificity in early detection and recurrence diagnosis. The 5-year survival rates of the ovarian cancer at stage I and II are 90% and 70% separately [4]. Despite of the advance in the surgical treatment and adjuvant therapy, the 5-year survival rate of the ovarian cancer at advanced stage is less than 30% [4]. Early detection of ovarian cancer is related to the obvious higher 5-year survival rate, and early diagnosis of recurrence is also important. Several new approaches such as the TP53 autoantibody, DNA methylation assays, microRNA algorithms, Pap-like cytologic analysis have been reported to improve sensitivity of the early detection of ovarian cancer [5]. However, it is urgent but still challengeable to develop new detection method with higher sensitivity for all stage of ovarian cancer.

Recently researchers found that the early-stage tumors can shed into cancer cells into bloodstream and cause metastasis [6]. Cells enter into the peripheral bloodstream through intravasation from primary tumors, recurrences, or metastases called circulating tumor cells which can be used as diagnostic or prognostic biomarkers for solid tumors [7]. The CTCs are rare in peripheral blood, and the detection methods need high sensitivity and specificity. In recent years, immunomagnetic separation, microfluidic separation, filter-based methods, and ligand-targeted PCR were reported in the detection of CTC [8–11]. To date, cell search system of Janssen Diagnostics is the only US Food and Drug Administration (FDA)-approved CTC detection method, which can be used to monitor patients with metastatic breast, colorectal, and prostate cancers [12–14]. The detection of CTC in ovarian cancer provides a noninvasive diagnostic method and has an advantage when it is difficult for biopsy. However, the detection rate of CTC in early stage of ovarian cancer is still low. The new methods to detect CTC with higher sensitivity are still needed. If we can easily detect CTC in ovarian cancer patients, it can be useful both in the early tumor detection, monitoring recurrence, and treatment effect.

Graphene, a two-dimensional semiconductor was isolated by Andre Geim and Kostia Novoselov in 2004 [15]. Recently, graphene-like 2D materials have been widely applied in sensing and bio-sensing, energy conversion and storage, catalysis, composites and coatings, electronics and biomedical field [15]. Graphene sensor is a promising candidate to detect cancer biomarkers due to its unique structure and excellent electrical performance, which had been developed to detect carcinoembryonic antigen, prostate specific antigen, carbohydrate antigen 19–9 and 15–3 [16–19]. Compared to conventional graphene-based biosensors fabricated on rigid SiO_2_/Si substrates by conventional photolithography, electrode evaporation, lift-off and sensor package process using precious facilities, it is significant to develop a low-cost and facile approach to fabricate flexible graphene-based biosensors with high sensitivity and rapid detect speed.

To address such issues, herein, we develop a novel and facile approach to fabricate graphene-based flexible biosensor on PET substrate. Two electrodes were directly fabricated on graphene/PET by using silver paste, and the cell pool was directly constructed by using silicone gel; this flexible biosensor can be made by hand in any laboratories without needing photolithography process and precious facilities. Surprisingly, our graphene-based flexible biosensors demonstrate highly sensitive and can rapidly detect for ovarian cancer cells. As far as we know, there are not any reports about flexible graphene-based biosensors for detection of ovarian cancer cells yet.

## Materials and Methods

### Growth and Transfer of Graphene Film

In this work, the graphene film was grown on the surface of Cu foil (Alfa Aesar, No. 13382) by chemical vapor deposition (CVD) [20]. Firstly, the surface oxide of Cu foil was removed by 20% hydrochloric acid solution for 5 min; then, the Cu foil was cleaned by de-ionized water for several times and then, dried with nitrogen flow. The cleaned Cu foil was put on a quartz boat and put into the quartz tube of CVD furnace. The furnace chamber was pumped down to 1 × 10^–2^ Pa. The furnace temperature was increased up to 1000 °C for 20 min with50 sccm of 99.999% H_2_, and then, 50 sccm of 99.999% methane was introduced into the tube for growth of large-area graphene film for 20 min. Finally, the CVD furnace was cooled down room temperature with CH_4_/H_2_ gas flow.

The large-area graphene/Cu foil was cut into many desired pieces. Then, the PMMA was spin-coated on the surface of graphene/Cu foil, forming the PMMA/graphene/Cu sandwich-like structure. Subsequently, the underlying Cu foil was etched with 1 M FeCl_3_ solution. The PMMA/graphene was cleaned in DI water for 30 min and then transferred onto the PET substrate. Finally, the PMMA was removed by acetone, and graphene/PET sample was obtained.

### Fabrication of Graphene-Based Biosensors

The fabrication procedure of graphene-based biosensors is described as follows. Firstly, about 1 cm × 2 cm graphene film on Cu foil was transferred onto 1 cm × 2 cm PET substrate by PMMA-assisted wet transfer method. Then, two electrodes were fabricated near around the center of graphene/PET film by using silver paste. Finally, in order to test the electrical response of the cancer cell solution, a cell pool with several millimeter length and width, and about 1 mm-height was constructed by silicone gel at the edge of the electrode. After the silicone gel of the cell pool is completely solidified, one can use Agilent 4155B semiconductor analyzer to check whether the graphene biosensor can work normally.

### Culture of SKOV3 Ovarian Cancer Cells

SKOV3 ovarian cancer cell series (provided by the public laboratory of the Second Affiliated Hospital of West China) were cultured in RPMI-1640 (Transgene, France) complete medium containing 10% calf serum (MRC, USA) under the condition of 5% CO_2_ and 37 °C.

### Preparation of Cell Solution and Electrical Measurement

The cancer cells were diluted to a specific concentration with cell culture medium. Take 50μL cell solution with pipette to the groove for measurement. The electrical signal was recorded by Agilent 4155B semiconductor analyzer.

## Results and Discussion

**Fig. 1:**
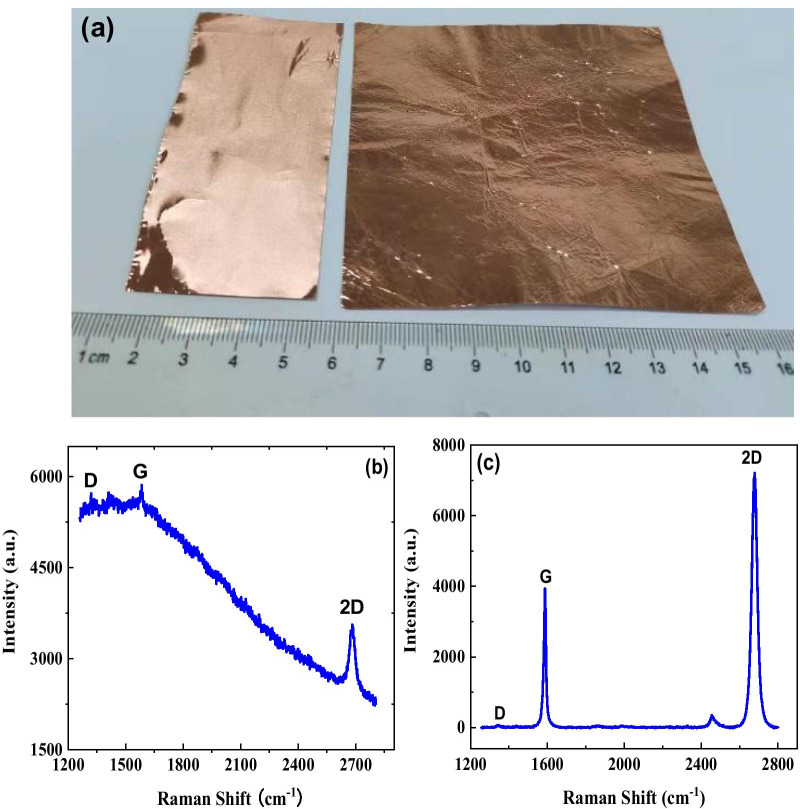
**a** Photograph of bare Cu foil (left panel) and graphene grown on Cu foil (right panel), **b** Raman spectrum of graphene/Cu, and **c** Raman spectrum of graphene/SiO_2_/Si

The photograph of 10 × 10 cm^2^ large-area CVD-grown graphene film on Cu foil is shown in Fig. 1 a. From Fig. 1a, one can observe that compared to bare Cu foil with bright metallic color, the color of graphene/Cu is a little bit darker. Corresponding Raman spectrum of graphene/Cu is shown in Fig. 1b. As shown in Fig. 1b, the Raman peaks at 1580 cm^−1^and 2680 cm^−1^ correspond to G and 2D peaks of graphene film. In order to further check the quality of graphene film, we have measured the Raman spectrum of monolayer graphene film transferred onto SiO_2_/Si substrate, as shown in Fig. 1c. One can observe that the ratio between *I*_G_ and *I*_2D_ is lower than 0.5, which confirms that the thickness of the graphene is monolayer; one can also observe that D peak is very low and nearly cannot be observed, which suggests that the quality of the graphene film is very high and the defects are very few.

**Fig. 2:**
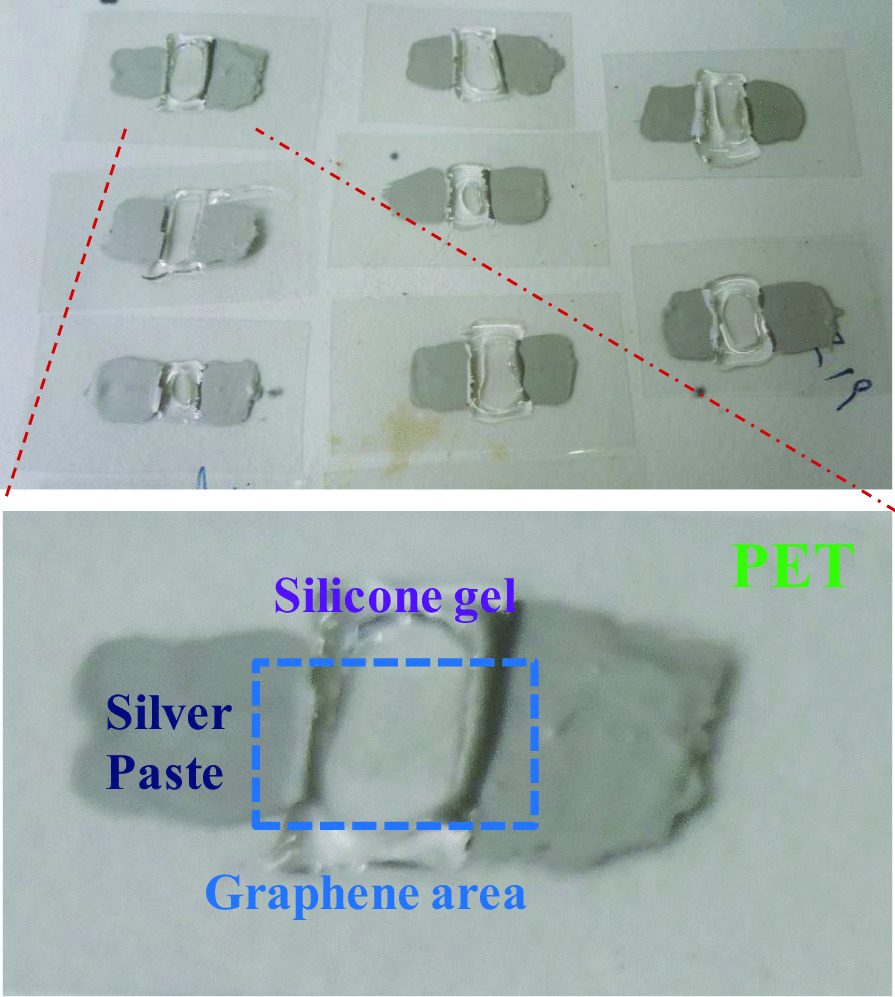
Photograph of graphene/PET biosensor

The photographs of flexible graphene biosensors on PET substrates are shown in Fig. 2. The cancer cell solution can be added into the cell pool, and the electrical signal of graphene biosensor can be obtained from two silver paste electrodes. The electrical response for cell culture medium, and CTC solution were measured.

**Fig. 3:**
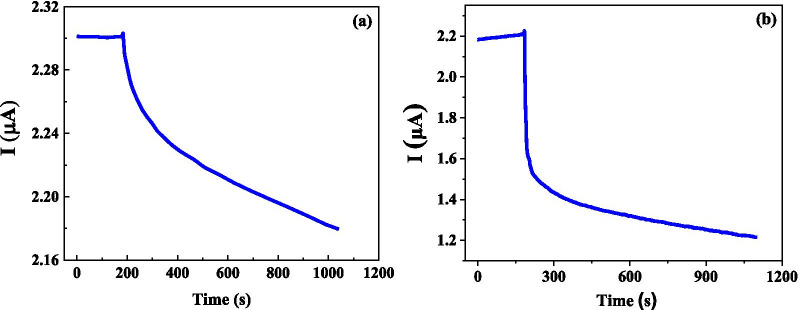
**a** Electrical response for bare cell culture medium, and** b** cancer solution with 30 cells

The time dependence of current response for such liquids is recorded at a fixed voltage of 0.01 V before and after putting them into the cell pool. As shown in Fig. 3, one can observe that before dipping such liquid, the current keeps constant; when such liquids were put into the cell pool, the current quickly decreases and then slowly keep a new balance. The response is defined as *η* = (*I*_0_ − *I*)/*I*_0_*100%, where *I*_0_ is the current just before dipping the liquid, and *I* is maximal (or minimal) value after dipping the liquid at some time. One can see that after one put such liquids, the resistance of grapheme increases. The electrical response for bare cell culture medium, and CTC solution before and after dipping solution 200 s are 2.96%, and 37.04%, respectively. Obviously, compared to bare cell culture medium, the electrical response for CTC solution even with 30 cells/ml is very significant, which suggests that the flexible graphene-based biosensor is very sensitive for cancer cell detection.

**Fig. 4:**
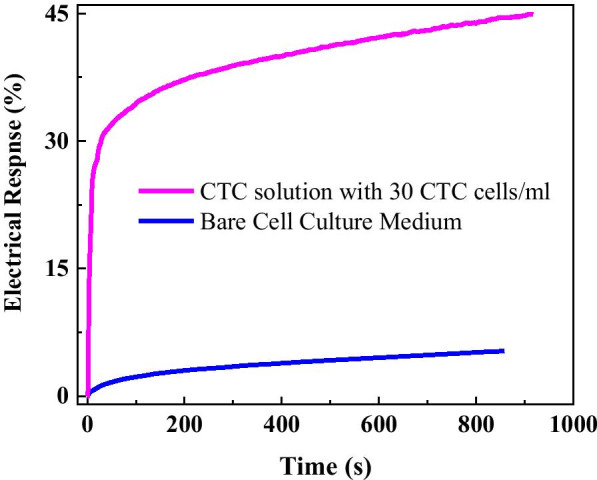
Time dependence of electrical response for cell culture medium and CTC solution

The time dependence of electrical signal for bare cell culture medium and CTC solution with 30 cancer cells/ml is further analyzed from Fig. 3. As shown in Fig. 4, one can observe that compared to that of bare cell culture medium, the electrical response for CTC solution (even as 30 cells/ml) is very sensitive and fast. After dipping the cell solution, it needs only 2.1, 2.0, 4.5, 7.5, 10.5, 28.5 s to reach response of 5%, 10%, 15%, 20%, 25%, 30%, while the response of corresponding cell culture medium only increases from 0.15 to 1.3%. That means, the flexible graphene biosensor is very suitable for rapid and sensitive detection within 5 s.

**Fig. 5:**
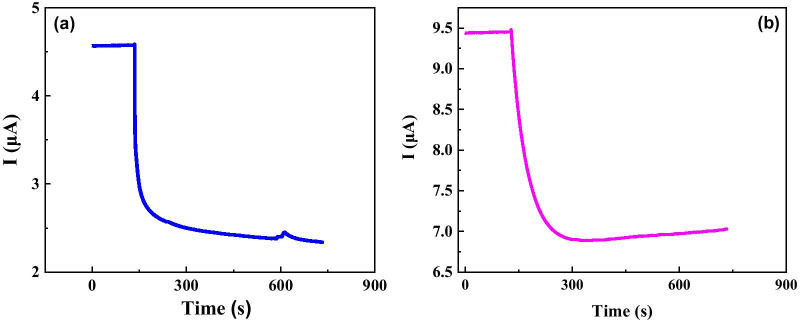
Time dependence of electrical response for different cancer cells: **a** SUDHL8, and **b** OCILYS

We further investigated the electrical response for two kinds of CTC cancer cells (SUDHL8 cells and OCILYS cells) with same concentrations of 10,000 (10 K)/ml. As shown in Fig. 5, the time dependence of current for two different cancer cells with a little bit different trend and a large difference in electrical response. That means, the graphene biosensor is promising to be used to identify different cancer cells.

**Fig. 6:**
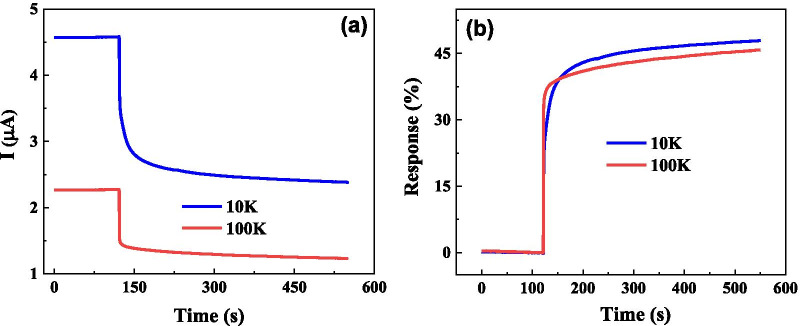
Time dependence of electrical current (**a**) and response (**b**) for SUDHL8 cancer cells with different concentrations of 10 K and 100 K cells/ml

The time dependence of electrical current and response for SUDHL 8 cancer cell solution with various cell concentrations of 10,000 (10 K)/ml and 100 K/ml has also been investigated. As shown in Fig. 6, one can observe that solution with lower cancer cell concentration solution shows higher current, which suggests that the cancer cell tends to be insulating and many more cells are not beneficial to conductive. The time dependence of response for two concentration solutions shows similar change trends and the response for lower concentration solution is a little bit higher than that of higher concentration solution. These results show that biosensor can be used to identify cancer solution with different concentrations.

As mentioned above, the results show that cheap and flexible graphene-based biosensor demonstrates different response for cell culture medium and cancer solution, different cancer cells and cancer cell solution with different concentrations, which suggests that such flexible graphene-based biosensor is promising to be used to detect and identify CTC ovarian cancer cells.

## Conclusion

In order to develop an efficient early detection method particularly for ovarian cancer, we develop a very simple graphene-based flexible biosensor on PET substrate. This flexible biosensor consists of a cell pool and two electrodes and compares the electric signal before and after adding cell solution, which shows high sensitivity and fast detection speed. It shows obvious different responses for cell culture medium and cancer solution, different cancer cells and cancer cell solution with different concentrations. Our work indicates that flexible graphene-based biosensor is promising to be used to sensitively and rapidly detect/identify CTC ovarian cancer cells.

## Data Availability

Authors can confirm that all relevant data are included in the article and its supplementary information files.

## Abbreviations

CA: Carbohydrate antigen
CTC: Circulating tumor cell
PET: Polyethylene terephthalate
FDA: Food and Drug Administration
CVD: Chemical vapor deposition
